# The Hospital Egg: Imports and the Contract System

**Published:** 1915-08-07

**Authors:** 


### THE EDITOR'S LETTER-BOX.

### The Hospital Egg: Imports and the Contract System.

*To the Editor of* The Hospital.

Sir,—As your correspondent, "Hospital," truly says, the all-important phase of this question is not whether pickled eggs are satisfactory (we say not), but whether there will be ample supplies all the winter. Our opinion is that we at least shall have little difficulty in keeping our customers going, unless, of course, something quite abnormal happens, in which case there would be difficulties in the supply of all other commodities, but none of us is contemplating such difficulties.

Now last year the stopping of the millions of eggs coming each week from Russia and neighbouring countries seriously affected all other classes. Russian eggs are largely used for cooking and other purposes where cheapness is essential (though not always advisable), and when they were unobtainable, and when the cold-stored Russians were finished, things were awkward. We, however, do not have to rely upon England; indeed, England cannot supply, in the most plentiful months, more than a small part of the quantity required. Ireland has done and is doing splendidly, improving every month, and sending tremendous quantities over here. We have been buying Irish eggs nearly twenty years, and very fine eggs come from there, particularly from the North of Ireland.

Canada and the States were not sending eggs here for some years, but now they are again sending, and very good the eggs are, though, of course not the best quality, the journey taking considerable time. Germany has been buying very large quantities of eggs from Russia, Italy, Austria, Galicia, Roumania, and other countries around them. Things are now different. For instance, the Dutch exporters are refusing German paper money! In several countries the egg industry is particularly well organised, and we are making arrangements for supplies that will, we have every reason to believe, pass our own very stringent tests for new-laid eggs.

This is a large market and supplies from new sources are constantly offered, there being plenty of enterprising people ready to pick up the opportunity neglected by our own people. We have established purchasing depôts throughout Lincolnshire and the Eastern Counties, Wales, and the North of Ireland, and have buying arrangements in other districts. We find this very advantageous in maintaining supplies, and the crisis through which we are passing has justified this looking ahead.

The Russian eggs now coming along, with those from other countries, are helping things very nicely. As a further reason for our optimism we may add that last December friends of ours having to do with military hospitals had much difficulty in obtaining adequate supplies. We offered our resources, as we had done in the early days of the war to the War Office. We started in mid-December supplying tens of thousands of new-laid eggs every week, and have since added several general hospitals to our list. We confess that the reason we had not gone whole-heartedly into the task of securing the orders of the general hospitals was the long credit so often taken, but we find that the committees and officials are open to reason, and that monthly payments can be arranged, which, of course, will ultimately benefit the institutions.

As to the advantages of the contract system, we confess we are not greatly enamoured of it. In normal times there may be advantages, though there are abuses, and we doubt if mere price is sufficient data to work upon. Not one of our customers other than hospitals have contracts, though we do give maximum prices on occasions, and it is found that where, as it should be, there is confidence in the firm supplying it is best not to have contracts, but to arrange the prices as they go along, and hotel managers are excellent business men. We, however, are willing to enter into contracts if that is preferred, though, of course, there are risks in selling so far ahead which we have to provide for in fixing prices, and we doubt whether buying by contract is really beneficial, though we fully realise that it takes some amount of courage for a hospital committee to place themselves in the hands of a firm. They, however, have the remedy of instantly withdrawing their patronage if there is anything at all unsatisfactory.—Yours faithfully, Wilkinson Bros.

37 Wilton Place, Knightsbridge, S.W.

